# Mycobacteria Bypass Mucosal NF-kB Signalling to Induce an Epithelial Anti-Inflammatory IL-22 and IL-10 Response

**DOI:** 10.1371/journal.pone.0086466

**Published:** 2014-01-28

**Authors:** Nataliya Lutay, Gisela Håkansson, Nader Alaridah, Oskar Hallgren, Gunilla Westergren-Thorsson, Gabriela Godaly

**Affiliations:** 1 Division of Laboratory Medicine, Department of MIG, Lund University, Lund, Sweden; 2 Division of Clinical Sciences, Department of Respiratory Medicine and Allergology, Lund University, Lund, Sweden; 3 Division of Vascular- and Respiratory Research Unit of Lung Biology, Department of Experimental Medical Science, Lund University, Lund, Sweden; Fundação Oswaldo Cruz, Brazil

## Abstract

The mechanisms by which mycobacteria subvert the inflammatory defence to establish chronic infection remain an unresolved question in the pathogenesis of tuberculosis. Using primary epithelial cells, we have analysed mycobacteria induced epithelial signalling pathways from activation of TLRs to cytokine secretion. *Mycobacterium bovis* bacilli Calmette-Guerin induced phosphorylation of glycogen synthase kinase (GSK)3 by PI3K–Akt in the signalling pathway downstream of TLR2 and TLR4. Mycobacteria did not supress NF-κB by activating the peroxisome proliferator-activated receptor γ. Instead the pro-inflammatory NF-κB was bypassed by mycobacteria induced GSK3 inhibition that promoted the anti-inflammatory transcription factor CREB. Mycobacterial infection did not thus induce mucosal pro-inflammatory response as measured by TNFα and IFNγ secretion, but led to an anti-inflammatory IL-10 and IL-22 production. Apart from CREB, MAP3Ks p38 and ERK1/2 activated the transcription factor AP-1 leading to IL-6 production. Interestingly, blocking of TLR4 before infection decreased epithelial IL-6 secretion, but increased the CREB-activated IL-10 production. Our data indicate that mycobacteria supress epithelial pro-inflammatory production by supressing NF-κB activation thereby shifting the infection towards an anti-inflammatory state. This balance between the host immune response and the pathogen could determine the outcome of infection.

## Introduction

Successful pathogen *Mycobacterium tuberculosis* (*M. tuberculosis*) use intricate strategy to evade the immune response. This pathogen invades the epithelial cells that cover the alveolar space of the lung and modulate or fine-tune the immune responses to produce a selective cytokine response [1]–[5]. The first phagocytes to be attracted to the infectious foci are the neutrophils [1], [2], [6], [7], followed by monocytes, and these leukocytes cooperate in the elimination of mycobacteria [8]. The extent of epithelial cytokine secretion may lead to tissue damage and breakdown of extracellular matrix, thus favouring bacterial persistence and facilitating mycobacterial transmission [9], [10]. However, perturbed defence in immune-compromised patients can tilt this balance leading to active disease [11]. These initial innate events, depending on the magnitude of the host immune responses, could thus determine the outcome of mycobacterial infection.

Epithelial cells express molecular pattern associated receptors, such as the Toll like receptors (TLRs) that interact with mycobacteria [12]. TLR2 expression increases upon mycobacterial infection of alveolar epithelium and blocking of TLR2 decreases cytokine responsiveness [4]. Mycobacteria express multiple ligands that bind to members of the TLR family, especially TLR2 and TLR4. Mycobacterial products, such as lipoarabinomannan (LAM) and the cell wall-associated and secreted 19-kDa glycolipoprotein, activate TLR signalling [4], [6], [13], [14]. TLR2 and TLR4 are also known to modulate the activation of peroxisome proliferator-activated receptor (PPAR)γ [15] that mycobacteria utilize to affect the NF-κB activation [16], [17]. Ligand binding to TLR initiates a signalling cascade through a MyD88-dependent and/or a MyD88-independent gene expression [18]. The MyD88-dependent activation leads to a pro-inflammatory cytokine response by the IRAK-NF-κB pathway, but also to chromatin remodelling by the MAPK kinases that regulates extracellular signal-regulated kinase 1/2 (ERK1/2), p38 proteins and c-Jun N-terminal kinase (JNK) [18]. The cytosolic domains of several TLRs bear also a conserved YxxM PI3K binding motif and phosphorylation of Akt, a downstream kinase activated by PI3K, is detected upon TLR stimulation [19]. Activation of Akt or p38 inactivates the glycogen synthase kinase 3 (GSK3) that is found further down the signalling pathway [20]. GSK3 is constitutively active in resting cells leading to the pro-inflammatory NF-κB transcription, but p38/Akt phosphorylation of GSK3 switches the transcriptional activity to cAMP response element-binding protein (CREB) [21]. TLR activation can thus either lead to a pro-inflammatory cytokine response by activation of NF-κB pathway, or an anti-inflammatory CREB-related cytokine response.

The initial events of mycobacterial infections are not clear. The first surface that the immobile bacterium will encounter after inhalation into the lungs would most likely be epithelial. Several groups have demonstrated that *M. tuberculosis* invades and survives within human type II alveolar epithelial cells [1], [22], [23]. Previous research revealed that the epithelia remain unresponsive to the infection until the third day, when the cells secreted a distinct pattern of cytokines [4], [5]. There are conflicting reports regarding the activation of NF-κB by pathogenic mycobacteria. In the present study, we have analysed mycobacteria induced epithelial signalling pathways from activation of TLRs to cytokine secretion. Our data indicate that mycobacteria avoid epithelial pro-inflammatory production by bypassing NF-κB activation thus balancing the infection towards an anti-inflammatory state.

## Materials and Methods

### Ethical Statement

The Swedish Research Ethical Committee in Lund (FEK 413/2008) approved the isolation of the bronchial material for primary cell cultures. Bronchial material for primary cell cultures was obtained from lung explant from healthy donors with irreversible brain damage and with no history of lung disease. Lungs were to be used for transplantation but could instead be included in this study as no matched recipients were available at that moment. Written consent was obtained from their closest relatives.

### Bacterial strains and growth conditions

*Mycobacterium bovis* bacillus Calmette-Guerin (BCG) Montreal strain containing the pSMT1 shuttle plasmid was prepared as previously described [24]. Briefly, the mycobacteria were grown in Middlebrook 7H9 culture medium, supplemented with 10% ADC enrichment (Becton Dickinson, Oxford, UK) and hygromycin (50 mg/l; Roche, Lewes, UK), the culture was washed twice with sterile PBS, and re-suspended in media again and then dispensed into vials. Glycerol was added to a final concentration of 25% and the vials were frozen at −80°C. Prior to each experiment, a vial was defrosted, added to 9 ml of 7H9/ADC/hygromycin medium, and incubated with shaking for 72 h at 37°C. Mycobacteria were then centrifuged for 10 minutes at 3000× *g*, washed twice with sterile PBS, and re-suspended in 2 ml of sterile PBS.

### Cell Culture

Bronchial tissue was dissected from lungs and kept in Dulbecco's Modified Eagle Medium supplemented with gentamicin, penicillin, streptomycin, Fungizone and 10% fetal calf serum (FCS) (all from Gibco, Paisley, UK) until further isolation. After removing intraluminal mucus and surrounding tissue, bronchi were digested in 0.1% Protease (Sigma St. Louis, MO) prepared in Minimum Essential Medium Eagle Spinner Modification (Sigma-Aldrich) supplemented with gentamicin, penicillin, streptomycin and Fungizone for 24 hours. Bronchial epithelial cells (HBEC) were recovered by repeated intraluminar rinsing with Dulbecco's Modified Eagle Medium supplemented with gentamicin, penicillin, streptomycin, Fungizone and 10% FCS. Cells were filtered through a 100 µm strainer (Falcon, Becton Dickinson) and seeded in cell culture flasks coated with 1% Collagen-1 (PureCol, Inamed Biomaterial, Freemont, CA) in Bronchial Epithelial Cell Growth Medium (Clonetics). The following day cells were thoroughly washed with a medium change every other day. Experiments were performed in passage 3 and 4.

### Infection and treatments of the cells

For the infection experiments, primary cells were grown in 6-well plates (2.0×10^5^ cells/well; Fisher Scientific, UK), infected with BCG (one bacterium per cell; 1 MOI) or phenol purified LPS (1 ng/ml; Sigma-Aldrich), lipoarabinomannan (LAM, 1 µg/ml, Lionex GmbH) or 19-kDa glycolipoprotein (1 µg/ml, Lionex GmbH) at 37°C for up to three days. For the blocking experiments, monoclonal mouse anti-human TLR2 or monoclonal mouse anti-human TLR4 antibodies (R&D Systems) 10 µg/ml were added to the epithelial cells 30 minutes before the addition of bacteria.

For cytokine analysis, the samples were collected after 0, 6, 24, 48 and 72 hours and for western blot analysis, the cells were detached by versene (140 mM NaCl, 2.4 mM KCl, 8 mM Na_2_HPO_4_, 1.6 mM KH_2_PO_4_, 0.5 mM EDTA, pH 7.2) and washed with PBS.

To investigate whether epithelial cells survive mycobacterial infection, we analysed cell viability by trypan blue exclusion assay according to manufactures instructions (Sigma Aldrich, Germany). For analysis of bacterial survival within the epithelial cells, infected epithelial cells were lysed in 300 µl of sterile distilled water for 15 minutes. 100 µl of the suspension was plated on Middlebrook 7H10 supplemented with 10% OADC Enrichment (Becton Dickinson, Oxford, UK) and grown for 3 weeks.

### Western Blot

The primary cells were washed with PBS containing 0.2 mM phenylmethylsulfonyl fluoride (PMSF), 1 µg/ml PepstatinA, 5 µg/ml Leupeptin (Sigma-Aldrich) and complete protease inhibitor cocktail (Roche Diagnostics, Mannheim, Germany) and lysed with modified Mammalian Protein Extraction Reagent (M-PER) solution (50 mM HEPES, 150 mM NaCl, 2 mM EDTA, 50 mM ZnCl, 1% NP-40, 0.1% deoxycholate, 0.1% SDS; Pierce) containing phosphatase (1∶10) and the complete protease inhibitor cocktail (1∶25). The cells were then placed on a shaker for 5 minutes, collected and centrifuged at 10,000×g for 5 minutes. Protein samples were used immediately for western blot analysis or stored at −80°C.

Protein levels were measured in cells treated with BCG and cells blocked for TLR2 or TLR4 with the NanoDrop™ 8000 Spectrophotometer using the Pierce 660 nm assay (Thermo Scientific). Medium alone, LPS, LAM and 19 kDa were used as controls. Protein samples were mixed with PBS, 4× NuPAGE LDS sample buffer (Life Technologies) and 1 M DTT and incubated at 90°C for 10 minutes followed by centrifugation at 218×g for 5 minutes. Equal amounts of protein (10 µg/well) were loaded on a NuPAGE 4%–12% Bis-Tris Gel (Life Technologies) and separated by sodium dodecyl sulfate-PAGE. A molecular weight marker (Novex® Sharp Prestained; Life Technologies) was loaded onto each gel for protein band identification. After separation, the proteins were transferred to a polyvinylidene difluoride (PVDF) membrane (Healthcare Amersham). The membrane was then blocked with either 5% dry-milk (Santa Cruz Biotechnology, Santa Cruz, CA) or with 5% bovine serum albumin (BSA; Santa Cruz Biotechnology) for 1 hour on a shaker at room temperature. Membranes were then incubated on a shaker overnight at 4°C with rabbit anti-human p-GSK-3α/β (1∶500; AF1590, R&D systems, Denmark), GSK-3α/β (1∶250; AF2157, R&D systems), p-CREB (1∶1000; #9198 Cell Signalling Technology, Inc., Danvers, MA), IκBα (1∶1000; #4812 Cell Signalling Technology), ERK1/2 (0.1 µg/mL, AF1018 R&D systems), PPARγ (1∶1000; NBP1-61399 Novus Biologicals), GAPDH (1∶500; sc-25778 Santa Cruz Biotechnology) or mouse anti-human NF-κB p65 (1∶200; sc-8008 Santa Cruz Biotechnology, Heidelberg, Germany), or β-actin (1∶10.000; Sigma-Aldrich) primary antibody. Incubation was followed by washing 3×5 minutes with Tris-buffered saline (TBS)-Tween 20 and 1×5 minutes TBS. The membrane was then incubated with goat-anti-rabbit IgG HRP (1∶2000; Santa Cruz Biotechnology) IgG secondary antibody or with rabbit anti-mouse IgG_1_ HRP (1∶4000; Dako) secondary antibody for 2 hours on a shaker at room temperature followed by washing with TBS-Tween 20 and TBS. The housekeeping protein GAPDH and β-actin were used to confirm equal loading on the wells. The membrane was developed using Amersham ECL Plus Western Blotting Detection Reagents (GE Healthcare, Little Chalfont, UK) and GelDoc equipment (Bio-Rad Laboratories). Blot intensity was quantified using ImageJ software 28 and normalized against GAPDH or β-actin. If required, membranes were stripped with Restore Western Blot Stripping Buffer (Pierce, Rockford, IL), blocked and re-probed with new antibodies.

### Phospho-kinase array

Protein phosphorylation was examined with the Proteome Human Phospho-Kinase Array Kit (Proteome Prolifer Array, R&D Systems, Abingdon, Oxford, UK), which is a membrane based sandwich immunoassay. The assay was performed according to the manufacturers' instructions. Briefly, total cell extracts were prepared from stimulated near-confluent cultures of normal human primary epithelial cells grown in 6-well plates. Untreated cells were used as control. The cell extracts containing 500 µg of total protein were incubated with the Human Phospho-Kinase Array. The proteins present in a lysate sample were captured by discrete antibodies printed in duplicate across the nitrocellulose membranes. The array was washed 3× with 1X Wash Buffer for 10 minutes on a rocking platform shaker to remove unbound proteins. Washing was followed by incubation with a cocktail of biotinylated detection antibodies (monoclonal anti-human of phosphorylated Akt (S473), Akt (T308), AMPK alpha1 (T174), AMPK alpha2 (T172), beta-Catenin, Chk-2 (T68), c-Jun (S63), CREB (S133), EGF R (Y1086), eNOS (S1177), ERK1/2 (T202/Y204, T185/Y187), FAK (Y397), Fgr (Y412), Fyn (Y420), GSK-3 alpha/beta (S21/S9), Hck (Y411), HSP27 (S78/S82), HSP60, JNK pan (T183/Y185 T221/Y223), Lck (Y394), Lyn (Y397), MSK1/2 (S376/S360), p27 (T198), p38 alpha (T180/Y182), p53 (S15), p53 (S392), p53 (S46), p70 S6 Kinase (T421/S424), PDGF R beta (Y751), PLC gamma-1 (Y783), PRAS40 (T246), Pyk2 (Y402), RSK1/2/3 (S380/S386/S377), Src (Y419), STAT2 (Y689), STAT3 (S727), STAT3 (Y705), STAT5a (Y694), STAT5a/b (Y694/Y699), STAT5b (Y699), STAT6 (Y641), TOR (S2448), WNK-1 (T60), Yes (Y426) and subsequent application of streptavidin-HRP conjugate. The signals were detected with the ECL Plus Western Blotting Detection System (GE Healthcare). Developed signals were analyzed using ImageJ 1.45s analysis software.

### Immunofluorescence microscopy

Expression of p-CREB and NF-κB in primary cells was detected by immunofluorescence staining. After blocking and infection for 72 hours the cells were fixed with 3.7% formaldehyde and then permeabilized in a mixture of PBS, 0.25% Triton X-100 and 5% fetal calf serum (FCS) for 30 minutes shaking at room temperature. Specimens were then incubated for 2 hours shaking at room temperature with PBS, 5% FCS, and the primary anti-rabbit p-CREB-1 (Ser133) or anti-mouse NF-κB p65 antibodies (1∶50; Santa Cruz Biotechnology). The cells were washed two times with PBS at 400×g for 5 minutes and then incubated with goat anti-rabbit or rabbit anti-mouse secondary antibody (1∶100; Invitrogen) in PBS and 5% FCS for 1 hour (shaking in dark) in room temperature. After additional washing the cells was stained with 1 µg/ml of 4′, 6-diamidino-2-phenylindole (DAPI) dissolved in PBS for 5 minutes in dark and then washed again with PBS. Finally the slides were mounted in fluoromount Aqueous Mounting Medium (Sigma Aldrich, F4680). The slides were examined with an inverted Nikon microscope (Nikon Diaphot 300) equipped with a 100 W mercury lamp (Osram, Berlin, Germany) and Ploempac with the filter set for fluorescein isothiocyanate and BioRad MRC 1024, controlled via LaserSharp (version 5.2 for PC/Windows) and further examined with the LSM 510 DUO confocal equipment with LSM software version 4.2 SP1 (Carl Zeiss, Jena, Germany). Sections incubated without primary or secondary antibody were used as negative controls to verify the lack of auto-fluorescence and unspecific secondary antibody staining.

### ELISA

IL-6 (D6050), TNFα (DTA00C), IFNγ (DIF50), IL-10 (D1000B) and IL-22 (D2200) secretion by the infected cells were quantified in supernatants by Human Quantikine ELISA Kits (R&D Systems, Oxon, UK) according to manufactures instructions. NF-κB (EK1111) and AP-1 (c-Jun, EK1041) were quantified with nuclear extraction kits containing ELISA-kit according to manufacturers instructions (Affymetrix Panomics, UK).

### Statistics

The statistical program used was SigmaStat, version 3.5, for Windows XP. The statistical difference between two groups was investigated by Mann-Whitney test. Multiple comparisons were done by one-way Analysis of Variance followed by Bonferroni test or Dunnett's test (***P≤0.001, ** P<0.01, *P<0.05, ns = non significant).

## Results

### Mycobacteria supress NF-κB and c-Jun

**Figure 1:**
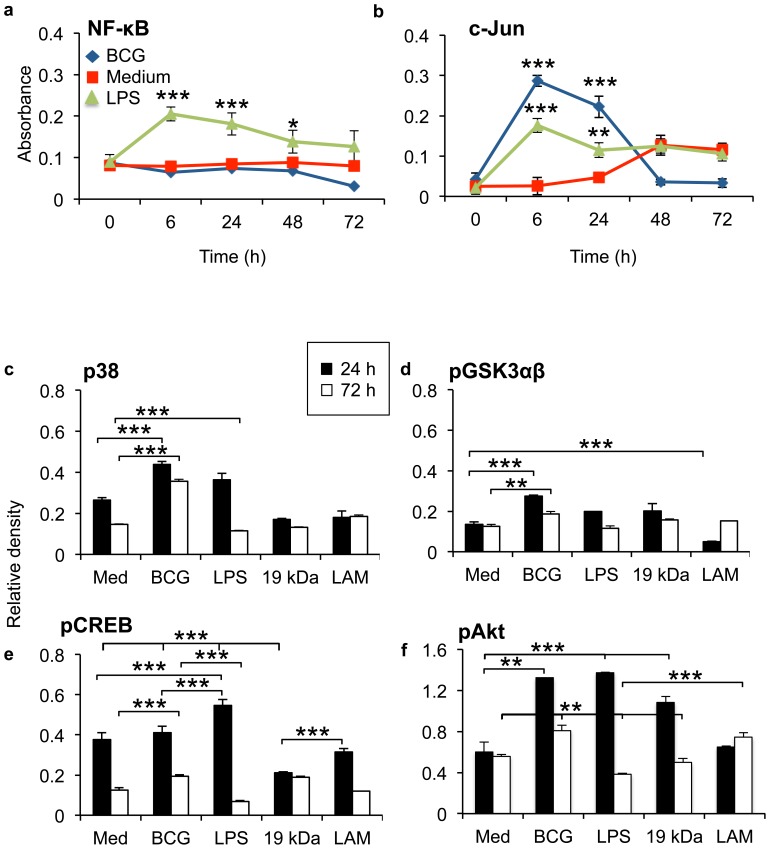
Mycobacteria bypass epithelial NF-κB signalling. (a) Infection of primary epithelial cells did not induce NF-κB activation quantified by ELISA, but an early activation of c-Jun proteins in epithelial cells was observed. (b) Epithelial GSK3αβ-pathway was analysed by Phospho-kinase array upon mycobacteria infection. In the beginning of infection, live mycobacteria, the virulence factors LAM and 19 kDa, and the TLR4 agonist LPS, induced comparable induction of p38, pAkt and pGSK3αβ. During the first 24 h, LPS induced higher increase of pCREB protein levels than mycobacteria (p = 0.0017). Third day of infection, mycobacteria significantly increased epithelial pCREB compared to medium control (p = 0.0357) or LPS (p = 0.0089). Epithelial stimulation with LAM induced an increase in pGSK3αβ and pAkt phosphorylation (p = 0.001 respectively p = 0.0196) during the later stages of infection compared to the early time-point. Generally, mycobacteria induced a more persistent increase of the investigated transcription factors three days after infection in primary epithelial cell than the controls LPS, 19 kDa and LAM. Data are presented as mean ± SEM of three separate experiments; **p<0.01 and ***p<0.001.

We used a low infection dose of 1∶1 (bacterium∶cell) [25], [26] and analysed alveolar nuclear extracts for NF-κB and c-Jun by ELISA. BCG at low MOI was shown to invade and survives in alveolar epithelial cells three days after infection without affecting epithelial viability (Figures S1 and S2). The TLR4 agonist LPS was used as a control. Infection of primary epithelial cells did not induce NF-κB activation during the three days of infection (Figure 1a). However, mycobacterial infection induced an early activation of c-Jun proteins that was suppressed two days after infection (Figure 1b). LPS induced an early NF-κB activation that was significantly higher than medium control and BCG up to 48 hours after addition to primary epithelial cells. Interestingly, BCG induced significantly higher c-Jun protein activation at 6 hours than LPS (p = 0.0177). We could confirm that BCG at low MOI invades and survives in primary epithelial cells [5], [27] three days after infection (Figure S3).

### Mycobacteria inactivates GSK3αβ signalling pathways

GSK3 consist of the isoforms α and β. The un-phosphorylated form of GSK3 promotes NF-κB activation, while phosphorylation of GSK3 by p38 and Akt promotes CREB anti-inflammatory activation. To investigate mycobacteria induced signalling pathways in primary epithelial cells, we analysed the GSK3αβ-pathway by Phospho-kinase array (Figure 1c–e). LAM and 19 kDa were used as controls for mycobacterial virulence factors and are known to signal through TLR2 [13], [14], while LPS is a known TLR4 ligand. In the beginning of infection, live mycobacteria, induced higher induction of p38 and pGSK3αβ than the virulence factor 19 kDa and the TLR4 agonist LPS (Figure 1c–d). Interestingly, mycobacterial virulence factor LAM significantly down-regulated pGSK3αβ after 24 hours of stimulation (Figure 1d). Mycobacteria and LPS induced higher increase of Akt than LAM and 19 kDa, but mycobacteria induced less pCREB protein levels during the first 24 hours, compared to LPS (p = 0.0017) or medium control (not significant) (Figure 1e–f). Three days after infection there was a significant increase of pCREB in mycobacteria infected epithelium compared to medium control (p = 0.0357) and LPS (p = 0.0089) (Figure 1e). Generally, mycobacteria induced a more persistent increase of the investigated transcription factors three days after infection in primary epithelial cell than the controls LPS, 19 kDa and LAM (Figure 1c–f). Interestingly, epithelial stimulation with LAM induced a late increase in pGSK3αβ and pAkt phosphorylation (p = 0.001respectively p = 0.0196) during the later stages of infection compared to the early time-point.

### Mycobacteria bypass NF-κB activation, but activated ERK1/2 and cFos

**Figure 2:**
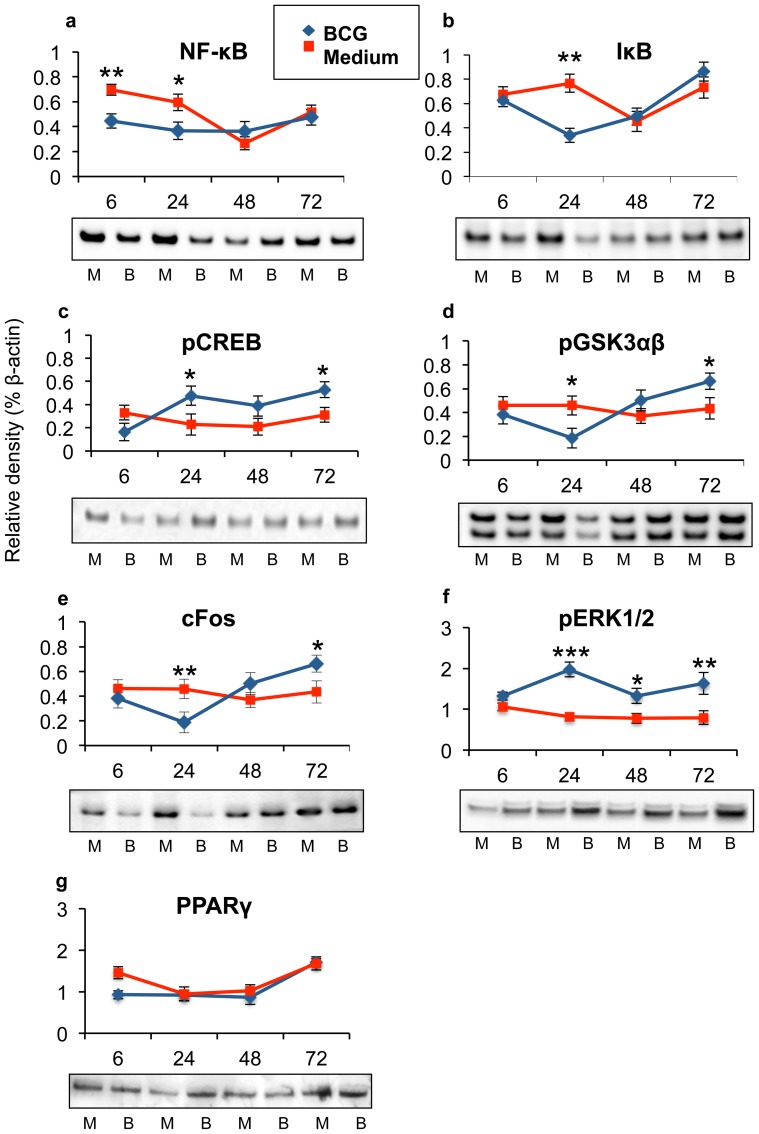
Mycobacteria modulate epithelial signalling pathways. Several molecules in the TLR-signalling pathway were analysed by Western blotting upon mycobacterial infection. (a–b) We could confirm that mycobacterial infection did not induce NF-κB- or IκB-activation. Mycobacterial suppression of primary epithelial (b) (p = 0.002) IκB and (d) (p = 0.0148) pGSK3αβ proteins were mostly pronounced at 24 hours of infection. The phosphorylated forms of (c) (p = 0.0163) CREB and (d) (p = 0.0248) GSK3αβ proteins reached highest levels 72 hours after infection. (e) Mycobacterial infection increased the Fos family of AP-1 proteins, as c-Fos protein levels significantly increased 72 hours after infection (p = 0.0038). (f) Mycobacteria induced two peaks of pERK1/2 protein levels, after 24 hours (p<0.001) and after 72 hours (p = 0.0034) of infection. (g) Epithelial cells express PPARγ protein, but mycobacterial infection did not significantly increase epithelial PPARγ amount. Data are presented as mean ± SEM of three experiments; *p<0.05, **p<0.01 and ***p<0.001.

To investigate mycobacteria induced epithelial signalling pathways further we analysed several molecules in TLR-signalling pathway by Western blotting. By comparing infected cells with un-infected cells during the investigated time-points, we could confirm that mycobacterial infection did not induce higher NF-κB- or IκB-activation (Figure 2a,b) than medium control during infection. Variations in suppression were observed during the time of infection. Mycobacteria supressed epithelial IκB and pGSK3αβ proteins at the beginning of infection (Figure 2b; p = 0.002 and Figure 2d; p = 0.0148 respectively), while the pGSK3αβ and pCREB proteins reached highest levels 72 hours after infection (Figure 2c,d; p = 0.0163 and p = 0.0248 respectively). Mycobacteria affected both GSK3 isoforms similarly. Interestingly, mycobacterial infection increased the Fos family of AP-1 proteins, as c-Fos protein levels significantly increased 72 hours after infection (Figure 2e; p = 0.0038). Mycobacteria induced two peaks of pERK1/2 protein levels, after 24 hours (p<0.001) and after 72 hours (p = 0.0034) of infection (Figure 2f). Mycobacteria were previously reported to induce PPARγ in order to modulate NF-κB responses [16], [17], but we could not observe that BCG significantly affected epithelial PPARγ protein concentration compared to medium control (Figure 2g). The actin loading controls are shown in the Figure S3.

### Mycobacterial infection controls epithelial cytokine production

**Figure 3:**
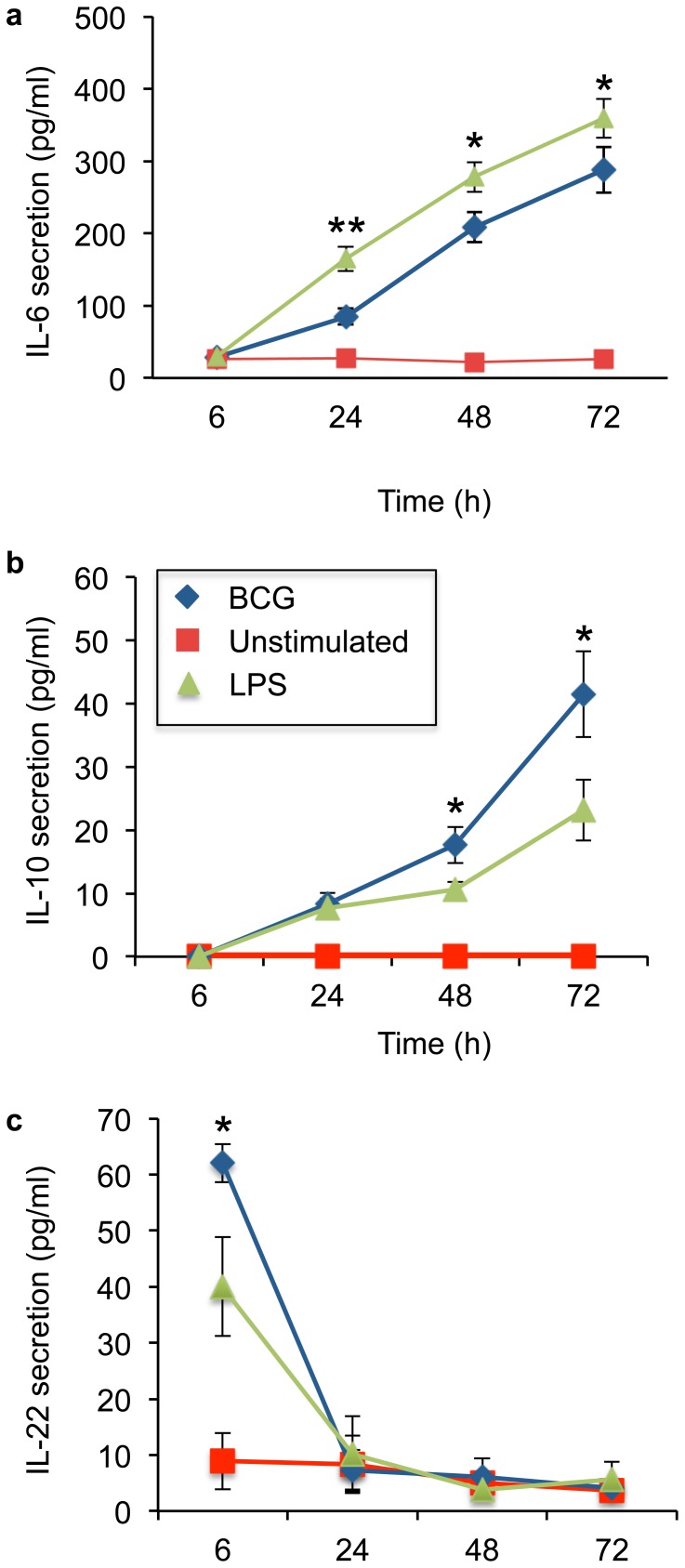
Controlled epithelial cytokine secretion. Mycobacterial control of induced transcriptional factors was analysed as epithelial cytokine secretion from six hours up to three days after infection. Infection induced a significant (a) IL-6 and (b) IL-10 secretion that peaked at 72 hours (p = 0.0425 and p = 0.0186 compared to LPS). (c) Mycobacterial infection of primary epithelial cells induced an early significant IL-22 secretion (p = 0.0463 compared to LPS) that ended 24 hours after infection. Data are presented as mean ± SEM of four separate experiments; *p<0.05 and **p<0.01.

Generally, the pro-inflammatory cytokines, such as IFNγ and TNFα, orchestrate innate and adaptive host immune responses, while anti-inflammatory cytokines, such as IL-10 and IL-22, confine the inflammation and postpone the generation of adaptive immunity [28]. Mycobacterial control of induced transcriptional factors was analysed as epithelial cytokine secretion from six hours up to three days after infection. Infection induced a significant IL-6 and IL-10 secretion that peaked at 72 hours (Figure 3a,b). In contrast, mycobacterial infection induced an early significant IL-22 secretion from primary epithelial cells that ended 24 hours after infection (Figure 3c). Mycobacterial infection did not induced epithelial TNFα or IFNγ secretion during the studied time interval (data not shown).

### Mycobacteria regulate TLR-induced inflammatory response

**Figure 4:**
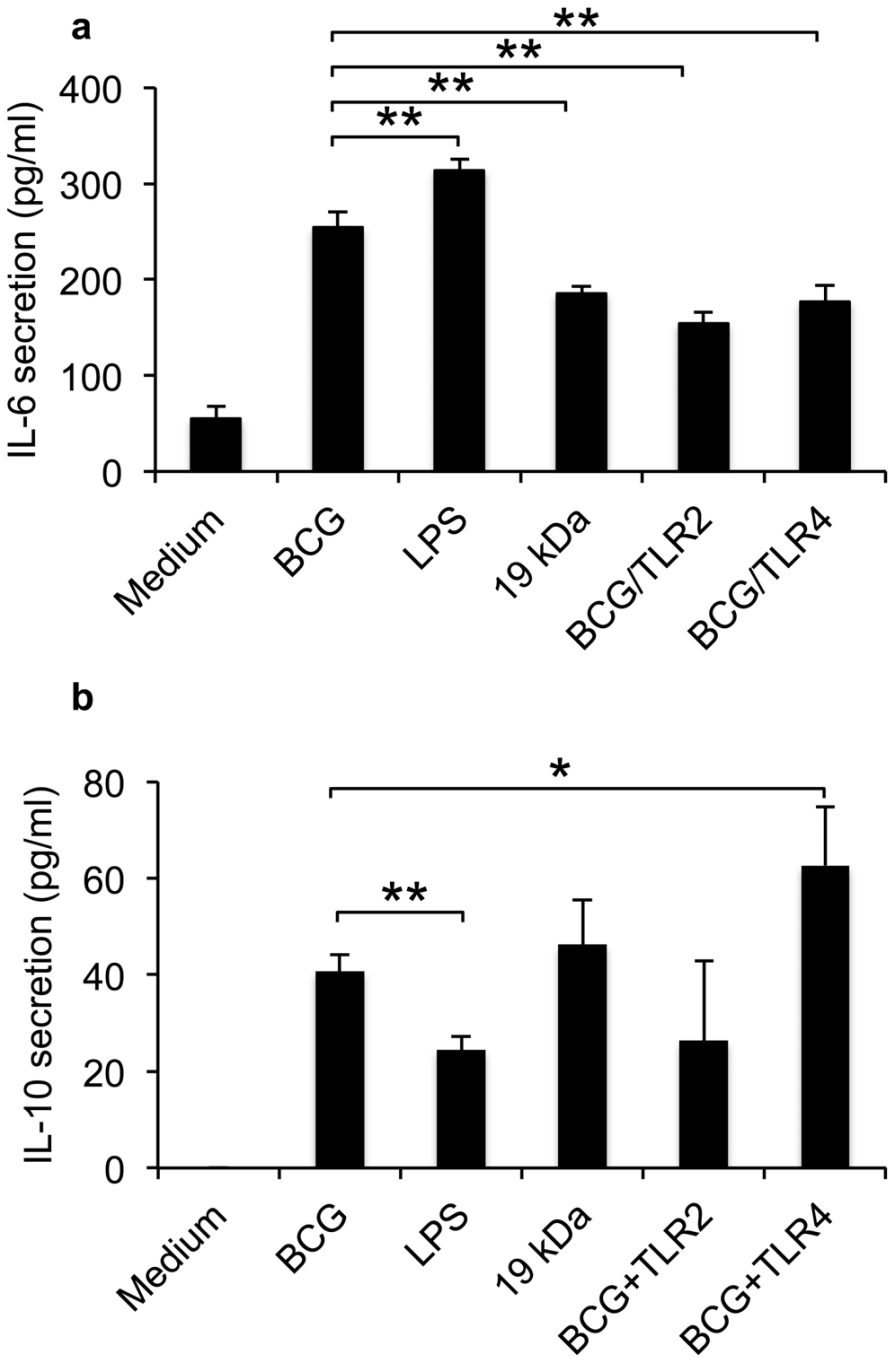
Mycobacterial regulation of TLR-induced cytokines. To determine the impact of TLR2 and TLR4 on mycobacteria induced pro- and anti-inflammatory cytokine production, the receptors were blocked prior to mycobacterial infection of the primary epithelial cells. (a) Blocking of TLR2 or TLR4 before infection decreased epithelial IL-6 secretion (p = 0.0011 and p = 0.0047 respectively) after three days. LPS induced a significantly higher IL-6 response than BCG (p = 0.0063), while 19 kDa induced a lower response compared to live mycobacteria (p = 0.0029). (b) Mycobacteria induced higher production of the anti-inflammatory IL-10 production than LPS (p = 0.0032) in human primary epithelial cells. Blocking of TLR4 prior to infection increased IL-10 secretion compared to unblocked infection (p = 0.0399). Blocking with TLR2 or addition of 19-kDa to the epithelial cells did not induce a significant change on epithelial IL-10 production compared to mycobacteria. Data are presented as mean ± SEM of three separate experiments; *p<0.05 and **p<0.01.

TLR-induced CREB activation is important for IL-10 production [21]. To determine the impact of TLR2 and TLR4 on mycobacteria induced pro- and anti-inflammatory cytokine production, the receptors were blocked prior to mycobacterial three-day infection of the primary epithelial cells (Figure 4). Antibody blocking of TLR2 or TLR4 before infection decreased epithelial IL-6 secretion (p = 0.0011 and p = 0.0047 respectively) (Figure 4a). The blocking of TLR2 or TLR4 did not affect alveolar survival during infection (Figure S1b). LPS induced a significantly higher IL-6 response than BCG (p = 0.0063), while 19 kDa induced a lower response compared to live mycobacteria (p = 0.0029). Mycobacteria induced higher production of the anti-inflammatory IL-10 production than LPS (p = 0.0032) in human primary epithelial cells (Figure 4b). Blocking of TLR4 prior to infection increased IL-10 secretion compared to unblocked infection (p = 0.0399). Blocking with TLR2 or addition of 19-kDa to the epithelial cells did not induce a significant change in epithelial IL-10 production compared to mycobacteria.

### Mycobacteria regulates CREB through TLRs

**Figure 5:**
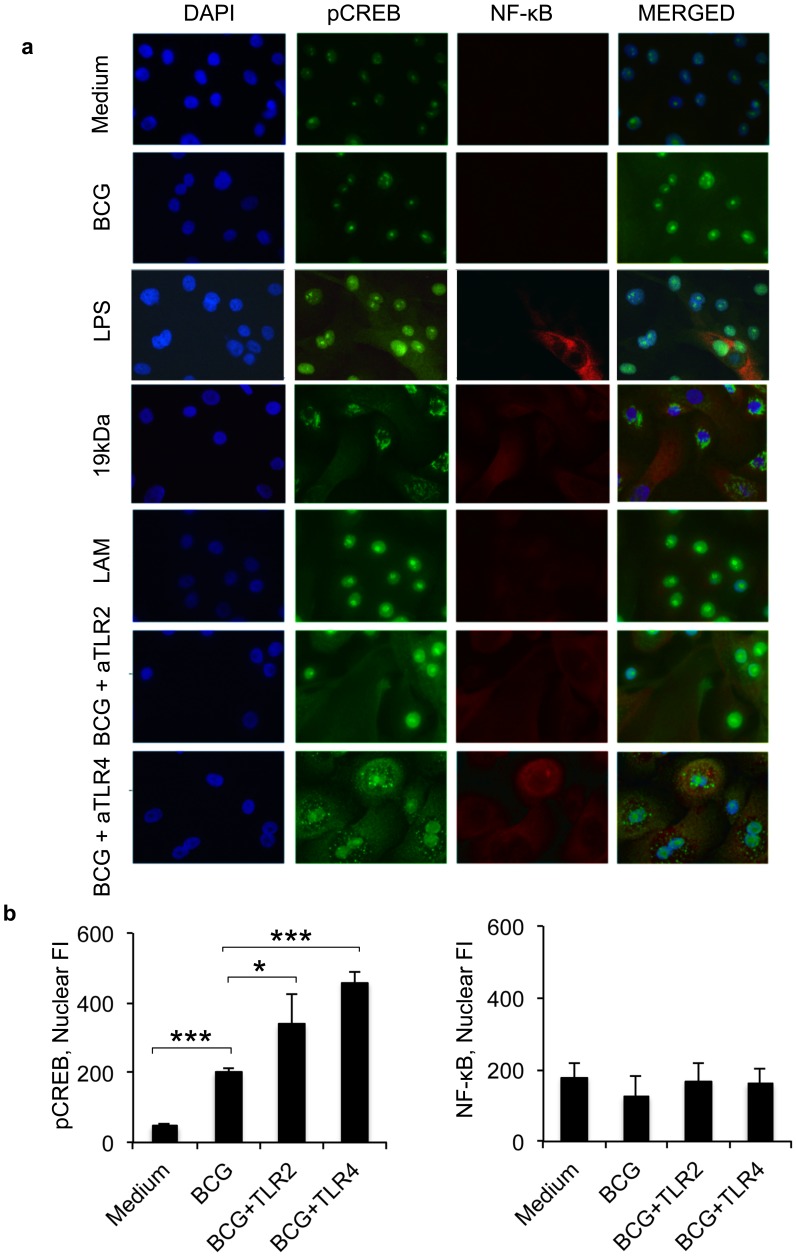
TLR4 blocking results in cytoplasmic CREB aggregation. Mycobacterial modulation of TLR signalling pathways was confirmed by immuno-fluorescence staining of pCREB and NF-κB expression in primary epithelial cells. (a) Mycobacterial infection increased nuclear pCREB protein levels compared to unstimulated cells, while the expression of NF-κB did not increase. Blocking of TLR4 before mycobacterial infection resulted in a granular cytoplasmic pCREB distribution, similar to pCREB aggregation in 19kDa-stimulated cells. TLR2 blocking and LAM treatment induced similar pCREB distribution as live mycobacteria. (b) The results were further analysed by LSM software. Mycobacterial infection increased significantly epithelial (p<0.001) pCREB expression as detected by confocal immuno-fluorescent microscopy, but NF-κB expression was not affected. Blocking of TLR2 or TLR4 before mycobacterial infection increased pCREB expression even further (p = 0.0187 and p 0.001 respectively) compared to unstimulated cells, but NF-κB expression was not affected. Original magnification × 300. Data are presented as representative images or mean ± SEM of three separate experiments; *p < 0.05 and ***p < 0.001.

Mycobacterial infection was previously shown to increase epithelial TLR2 and TLR4 [4]. The impact of TLR2 and TLR4 were analysed by immuno-fluorescence staining of pCREB and NF-κB expression in primary cells (Figure 5a). Mycobacterial infection increased nuclear pCREB protein levels compared to unstimulated cells, while the expression of NF-κB did not increase. Blocking of TLR4 before mycobacterial infection resulted in a granular cytoplasmic pCREB distribution, similar to pCREB aggregation in 19 kDa-stimulated cells. TLR2 blocking and LAM treatment induced similar pCREB distribution as live mycobacteria. Epithelial treatment with mycobacterial virulence factor 19-kDa resulted in a granular cytoplasmic pCREB distribution, while LAM treatment induced similar pCREB distribution as live mycobacteria (Figure 5a). Further confirming our results, detection of epithelial pCREB by confocal immuno-fluorescent microscopy revealed that mycobacterial infection significantly increased pCREB expression (p<0.001), but NF-κB expression was not affected (Figure 5b). Blocking of TLR2 or TLR4 before mycobacterial infection increased pCREB expression even further (p = 0.0187 and p<0.001 respectively) compared to unstimulated cells, but NF-κB expression was not affected (Figure 5b).

### TLRs are involved in mycobacterial regulation of mucosal inflammation

**Figure 6:**
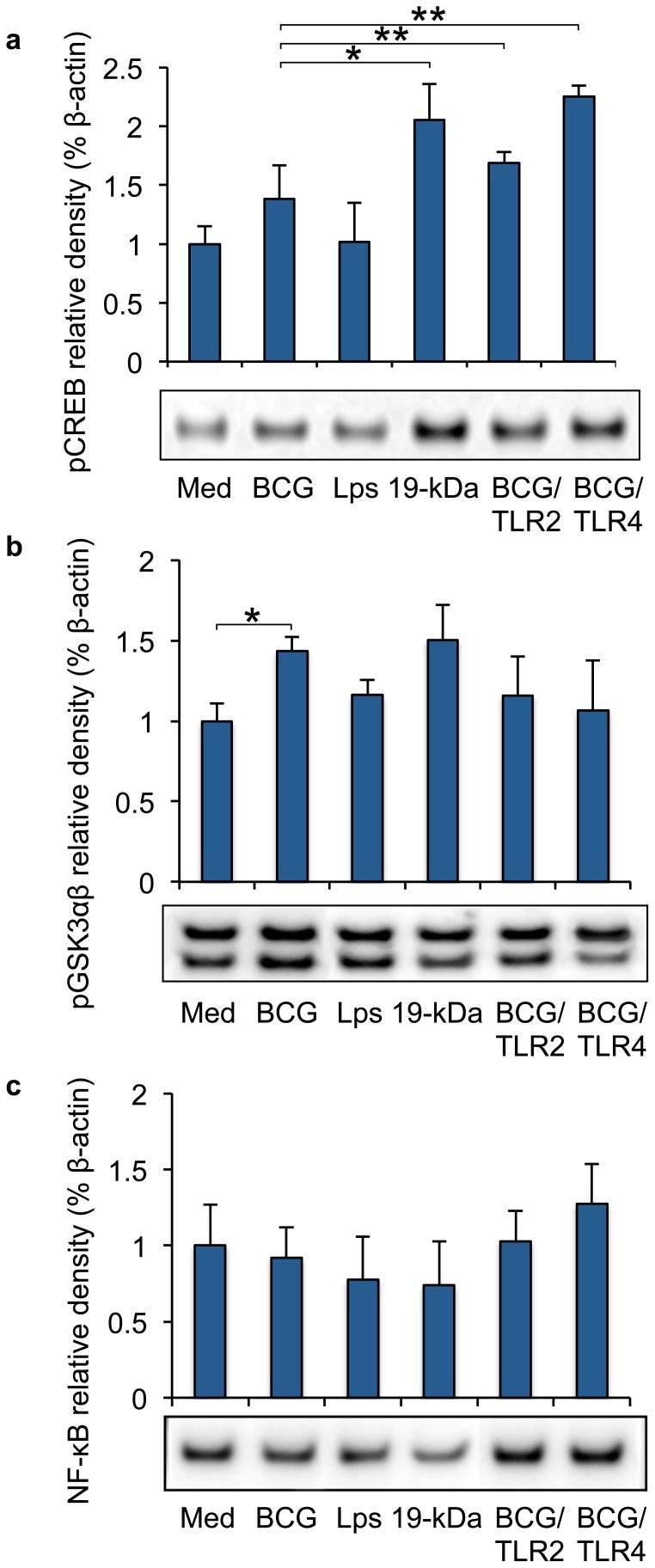
Mycobacteria control epithelial TLR responses. The impact of TLRs on mycobacterial modulated epithelial signalling was studied by Western blotting prior to infection and three days after infection. (a) Blocking of TLR2 (p = 0.0063) or TLR4 (p = 0.0047) prior to infection or stimulation with 19 kDa significantly increased epithelial pCREB production (p = 0.0163). (b) Blocking of TLRs or 19 kDa stimulation of epithelial cells had an non-significant impact on pGSK3βα expression. (c) Blocking of TLR2 or TLR4 before mycobacterial infection of primary epithelial cells non-significantly restored the NF-κB values to background levels. Data are presented as mean ± SEM of three separate experiments; *p<0.05 and **p<0.01.

To further determine the impact of TLR2 and TLR4 on mycobacteria induced cytokine production, the receptors were blocked prior to mycobacterial infection and the impact of modulated epithelial signalling was studied by Western blotting three days after infection. Blocking of TLR2 (p = 0.0063) or TLR4 (p = 0.0047) prior to infection or stimulation with 19 kDa significantly increased epithelial pCREB production (p = 0.0163) (Figure 6a). Blocking of TLRs or 19 kDa stimulation of epithelial cells had a non-significant impact on pGSK3βα expression (Figure 6b). Blocking of TLR2 or TLR4 before mycobacterial infection of primary epithelial cells non-significantly restored the NF-κB values to background levels (Figure 6c). The GADPH loading controls are shown in the Figure S4.

## Discussion

Functional NF-κB activation is essential for the maintenance of physiological immune homeostasis and protective host defence. We found that *Mycobacterium bovis* bacilli Calmette-Guerin bypassed NF-κB activation during the first days of infection. BCG is equipped with several genes coding for invasin/adhesin-like proteins [27], [29]–[33] and the mycobacterial adhesion heparin-binding haemagglutinin [34] is believed to be involved in invasion of human alveolar epithelial cells [35]. Activated NF-κB was recently shown to be essential for mycobacterial elimination, since blocking of this pathway prevented bacterial killing and allowed the bacteria to grow in macrophages [36]. To date, reported data regarding the activation of NF-κB by pathogenic mycobacteria are conflicting. *M. tuberculosis* was shown to supress NF-κB pathway in some studies [37], [38], induce a transient NF-κB activation in other studies and some studies observed activated NF-κB pathways under some conditions [39]–[42]. However, several bacteria are known to subvert the cell-intrinsic innate immunity by targeting NF-κB. *Salmonella*, *Shigella* and enteropathogenic *Escherichia coli* (EPEC) are known to supress the NF-κB pathway to counteract the host defences [43], [44]. Recent genetic studies revealed that EPEC suppression of host NF-κB signalling and NF-κB dependent anti-inflammatory cytokine production requires NleE, a type III-secreted effector that has homologues in *Shigella* and certain *Salmonella* species [45]–[47]. Recently, genome-wide screens identified previously unidentified gene products for *M. tuberculosis* persistence [48], but whether mycobacteria possess similar elements are not known.

Innate recognition of mycobacteria involves the activation of TLR2 and TLR4. Signalling through TLR activates the adaptor protein MyD88 leading to NF-κB signalling and the activation of ERK1/2, p38 and JNK [18]. Besides of MyD88, activation of TLRs triggers also PI3K activation leading to subsequent Akt phosphorylation. Akt and p38 phosphorylate the glycogen synthase kinase 3 (GSK3), which switches the transcription from the pro-inflammatory NF-κB to the anti-inflammatory CREB activation [21]. We observed that mycobacteria induced the MyD88 stimulated p38, ERK1/2 and AP-1 signalling. Interestingly, mycobacterial infection induced an early activation of the c-Jun family of AP-1 proteins in primary epithelial cells, and a late activation of the AP-1 protein Fos. Mycobacterial activation of PPARγ is known to supress NF-κB in macrophages [16], but we could not observe mycobacteria-induced PPARγ activation in primary epithelial cells. GSK3 regulates the transcriptional activity of CREB and NF-κB by competing for the limited amount of CREB-binding protein (CBP) [21]. TLR activation could therefore either lead to a pro-inflammatory cytokine response by activation of NF-κB pathway, or an anti-inflammatory CREB-related cytokine response. In this study, mycobacterial infection induced increased GSK3 phosphorylation, switching thus the transcriptional activity from NF-κB to CREB. Indeed, epithelial cells responded early to mycobacterial infection by secreting IL-6 and the anti-inflammatory IL-22, while the anti-inflammatory IL-10 increased two days after infection. The cytokine IL-6 is transcribed by CREB, C/EBP, STAT3 and AP-1 [49], [50], and can act as both pro- and anti-inflammatory in many chronic inflammatory diseases. IL-6 trans-signalling is critically involved in the maintenance of a disease state by promoting transition from acute to chronic inflammation [51]. In addition, IL-6 is required in the rapid expression of an initial protective IFNγ response during *M. tuberculosis* infection [52]. However, concomitant IFN production can tilt the anti-inflammatory qualities of IL-10 and IL-22 towards a pro-inflammatory state [53]. We could not observe epithelial IFNγ production, suggesting that the secreted IL-10 and IL-22 are produced to damper the inflammation. IL-10 modulates the anti-inflammatory mechanisms by targeting NF-κB thereby inhibiting cellular production of TNFα, which could be one of the mechanisms of NF-κB suppression that we observed in our study [54], [55]. Mycobacteria was previously reported to induce IL-10 secretion from neutrophils through the phosphorylation of p38 and Akt kinases [56]. Mycobacterial infection of *Il10* ^−/−^ mice show enhanced protection while showing no signs of aberrant host-mediated pathology, which perhaps reflects the slow disease progression [57], [58]. The role of IL-10 could be to limit mycobacterial clearance during the early immune response through the inhibition of IL-12p40 [59]. IL-22 is found in large amounts in pleura from TB patients [60] and this cytokine is primarily expressed by CD4^+^ T cells [61], but other leukocyte subsets also express this cytokine [62]. IL-22 acts through the IL-22 receptor complex expressed by epithelial cells and hepatocytes, where it promotes regeneration and protects against tissue damage [63], [64], but accumulating evidence suggests that IL-22 can be either pathogenic or protective depending on host conditions [65]. Using the TB mouse model, a recent study showed that neutralization of IL-22 did not have any effect on the lung bacterial burden or granuloma formation [66]. The mycobacterial vaccine strain used in our study did not induce TNFα or IFNγ secretion. Interestingly, recent studies support the IL-17-CXCL13 pathway rather than the IFNγ pathway as a new strategy to improve mucosal vaccines against tuberculosis [67]. We are currently investigating if alveolar epithelia induce IL-17 or CXCL13 upon mycobacterial infection.

Blocking of epithelial TLR4 before mycobacterial infection decreased the pro-inflammatory IL-6 secretion, but increased the anti-inflammatory IL-10 secretion. TLR4 blocking prior to mycobacterial infection resulted in a granular cytoplasmic pCREB distribution similar to the 19-kDa stimulated cells. We could not find any explanation of the cytoplasmic granular accumulation, but granular accumulation of pERK in cytoplasm was shown to alternate downstream signalling in Parkinson's disease [68]. Normally, signals that induce NF-kB activity usually lead to IkB phosphorylation by the IkB kinase (IKK) complex, and subsequent multi-ubiquitination and degradation of this protein via proteasome, allowing NF-kB dimers' translocation to nucleus [69]. We observed that TLR4 blocking induced cytoplasmic accumulation of NF-κB as well, although no increased NF-κB translocation to epithelial nuclei was detected.

Mycobacteria cause persistent infections by minimizing the degree of overt pathology, allowing long-term association with the host. We have observed that mycobacterial infection of primary epithelial cells supress NF-kB activation by increasing the inhibitory GSK3, thereby supporting the production to the anti-inflammatory cytokines IL-22 and IL-10. Production of anti-inflammatory cytokines is known to impair antigen presentation, which confines the inflammation and postpones the generation of adaptive immunity resulting in antigen-specific anergy. These events could lead to an impaired innate immune response by which mycobacteria create a safe haven for chronic infection and transmission to new hosts.

## Supporting Information

Figure S1 **Intracellular viability of mycobacteria.**
(TIF)

Figure S2 **Epithelial viability visualized by trypan blue exclusion assay three days after infection, with or without blocking of TLR2 or TLR4.**
(TIF)

Figure S3 **Actin loading controls (** **Figure 2** **).**
(TIF)

Figure S4 **GADPH loading controls (** **Figure 6** **).**
(TIF)
