# Supplementary figures and images for: Mycobacteria Bypass Mucosal NF-kB Signalling to Induce an Epithelial Anti-Inflammatory IL-22 and IL-10 Response
Source: PLoS One. 2014 Jan 28;9(1):e86466. doi: 10.1371/journal.pone.0086466 (PMC3904915; doi:10.1371/journal.pone.0086466)

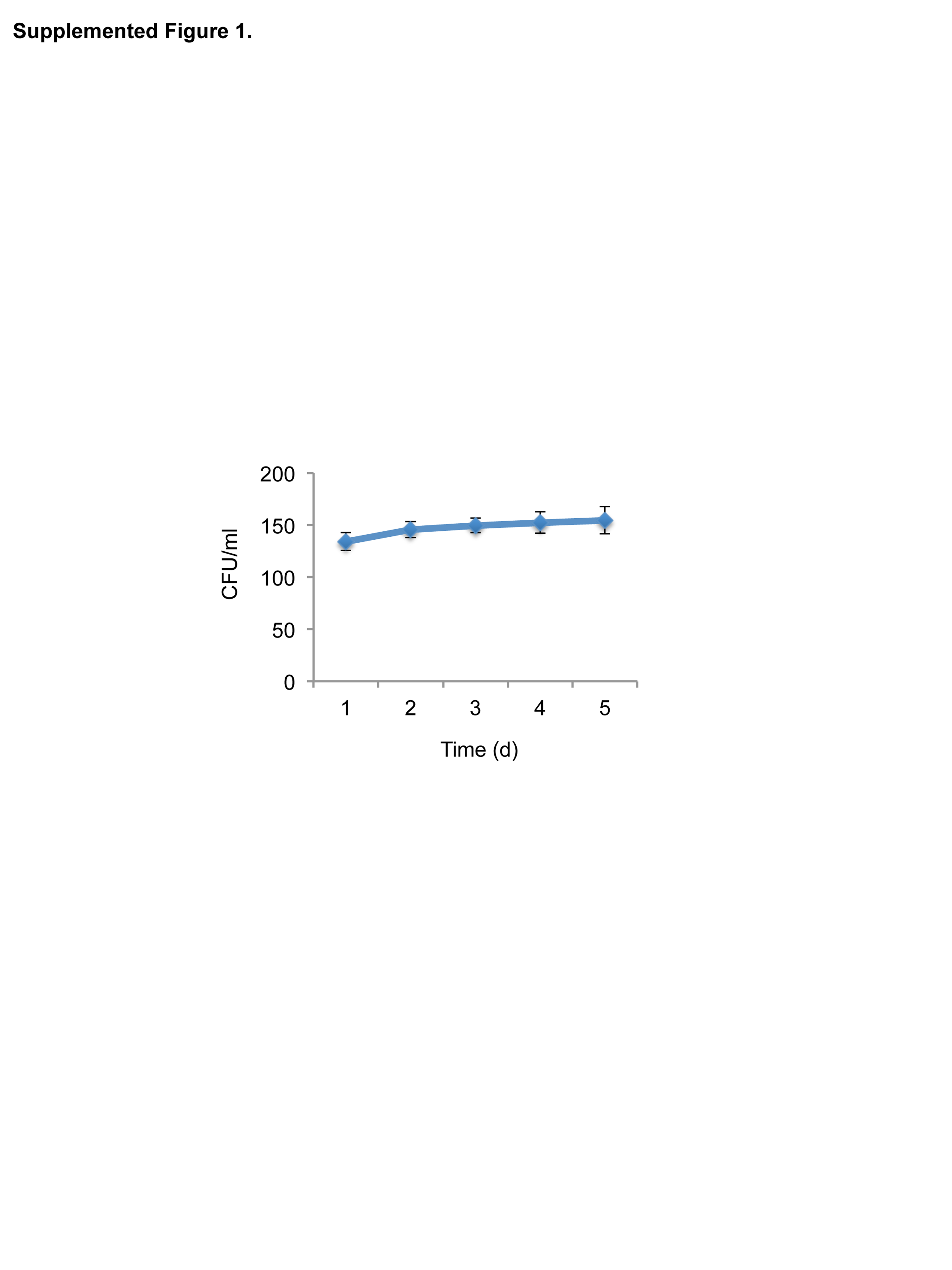

Supplement: Figure S1 — Intracellular viability of mycobacteria. (TIF) [file pone.0086466.s001.tif]

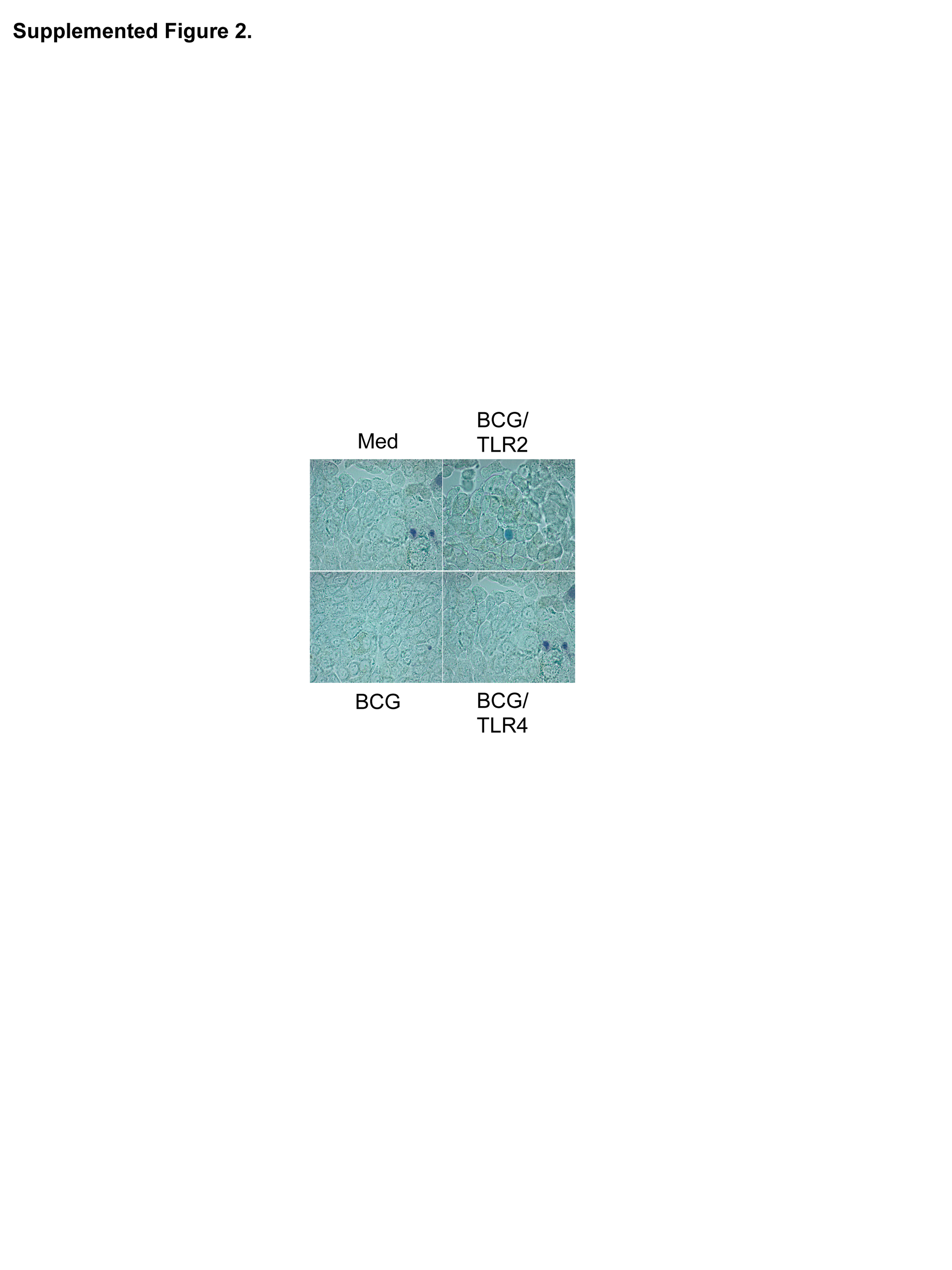

Supplement: Figure S2 — Epithelial viability visualized by trypan blue exclusion assay three days after infection, with or without blocking of TLR2 or TLR4. (TIF) [file pone.0086466.s002.tif]

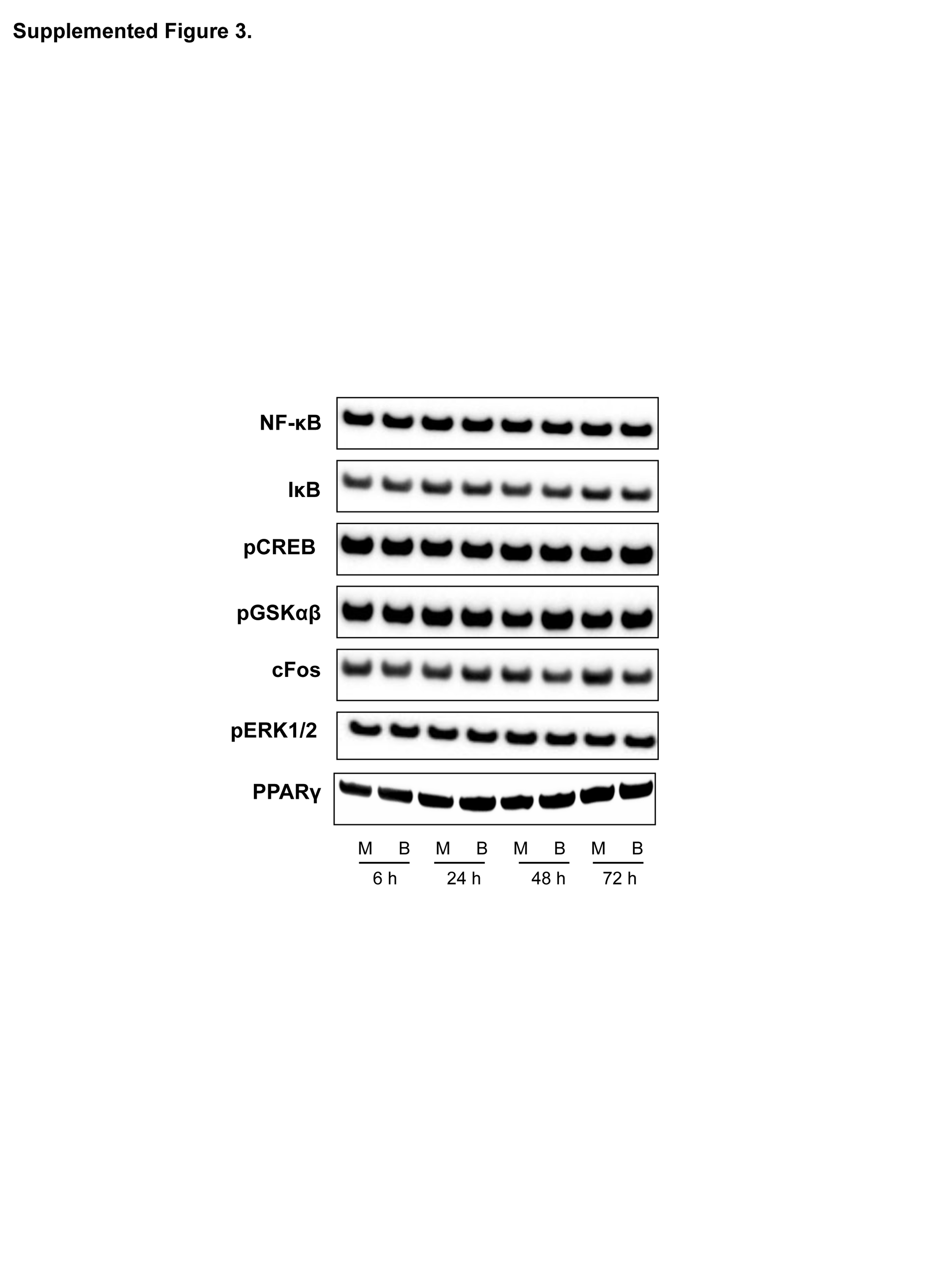

Supplement: Figure S3 — Actin loading controls ( Figure 2 ). (TIF) [file pone.0086466.s003.tif]

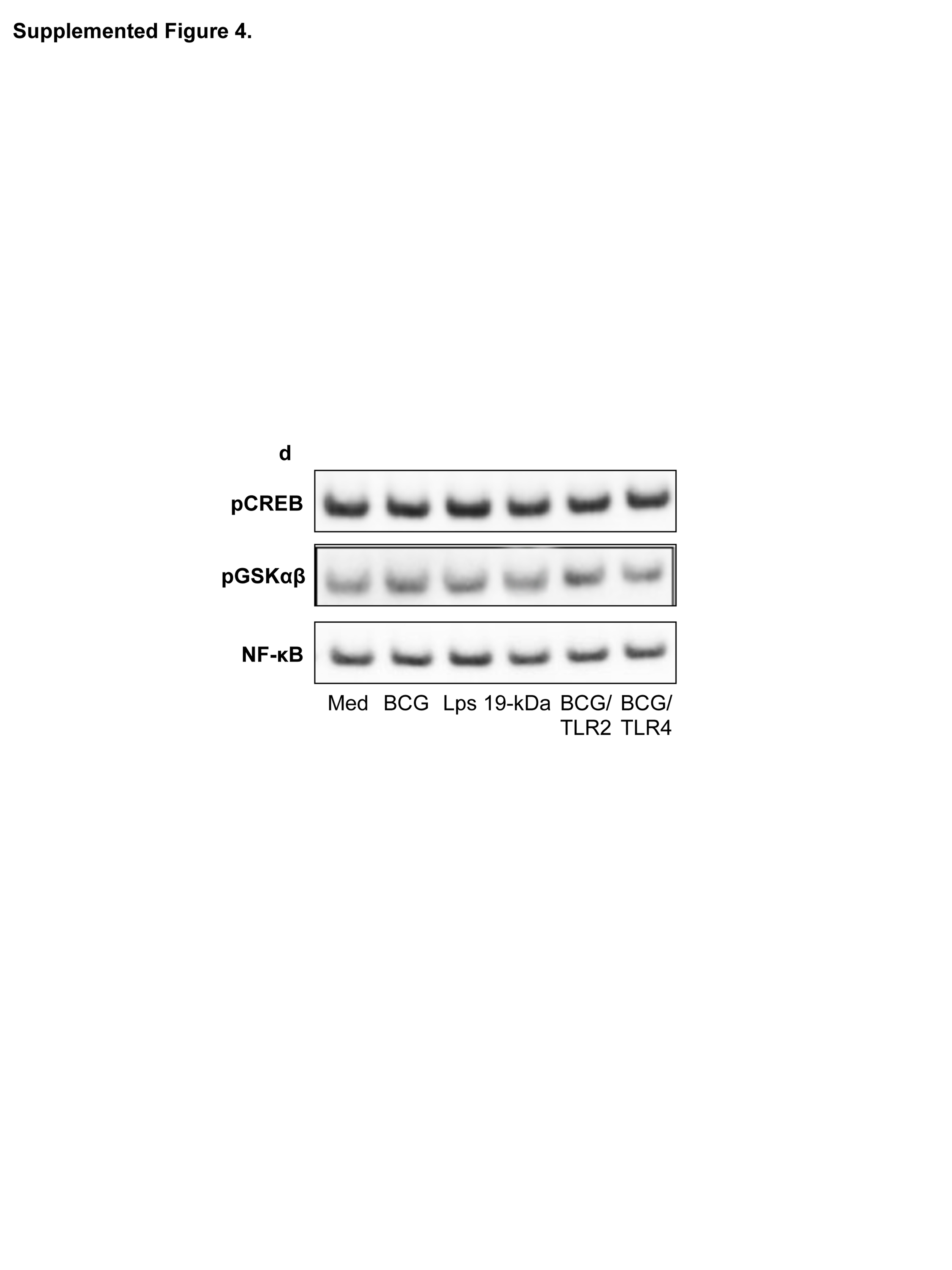

Supplement: Figure S4 — GADPH loading controls ( Figure 6 ). (TIF) [file pone.0086466.s004.tif]
